# Retraction Note: LncRNA PCAT6 regulates the progression of pituitary adenomas by regulating the miR-139-3p/BRD4 axis

**DOI:** 10.1186/s12935-024-03398-y

**Published:** 2024-06-14

**Authors:** Peng Zhao, Jianhua Cheng, Bin Li, Ding Nie, Hongyun Wang, Chuzhong Li, Songbai Gui, Yazhuo Zhang

**Affiliations:** 1 Department of Neurosurgery, Beijing Tiantan Hospital, Capital Medical University, No. 119 South Fourth Ring West Road, Fengtai District, Beijing, 100070 People’s Republic of China; 2 Beijing Tiantan Hospital, Beijing Neurosurgical Institute, Capital Medical University, Beijing, People’s Republic of China

**Retraction Note: Cancer Cell International (2021) 21:14**

10.1186/s12935-020-01698-7

The Editors-in-Chief have retracted this article. After publication, concerns were raised regarding high similarity between some of the data presented in this article and other publications. Specifically, Fig. 11a top right image appears highly similar to Fig. 3c top left image of [1, now retracted]. In addition, the ruler in Figs. 4a and 5a appears highly similar to that in Figs. 4a and 7a of [2, now retracted].

The Editors-in-Chief therefore no longer have confidence in the presented data.

All authors agree to this retraction.
